# Efficacy and acquired resistance of EGFR-TKI combined with chemotherapy as first-line treatment for Chinese patients with advanced non-small cell lung cancer in a real-world setting

**DOI:** 10.1186/s12885-021-08291-9

**Published:** 2021-05-25

**Authors:** Qianqian Wang, Wen Gao, Fangyan Gao, Shidai Jin, Tianyu Qu, Fan Lin, Chen Zhang, Jingya Zhang, Zhihong Zhang, Liang Chen, Renhua Guo

**Affiliations:** 1 Department of Oncology, the First Affiliated Hospital of Nanjing Medical University, 300, Guangzhou Road, Nanjing, 210029 Jiangsu China; 2 Department of Cell Biology, School of Basic Medical Sciences, Nanjing Medical University, 101Longmian Avenue, Nanjing, 211166 Jiangning China; 3 Department of Pathology, the First Affiliated Hospital of Nanjing Medical University, 300, Guangzhou Road, Nanjing, 210029 Jiangsu China; 4 Department of Thoracic and Cardiovascular Surgery, the First Affiliated Hospital of Nanjing Medical University, 300, Guangzhou Road, Nanjing, 210029 Jiangsu China

**Keywords:** Non-small cell lung cancer, First-line treatment, EGFR-TKI, Chemotherapy, Acquired resistance

## Abstract

**Background:**

To compare the benefits and explore the cause of acquired resistance of epidermal growth factor receptor tyrosine kinase inhibitor (EGFR-TKI) and its combination with chemotherapy in advanced non-small-cell lung cancer (NSCLC) patients harboring EGFR mutation in a real-life setting.

**Methods:**

This retrospective analysis included 117 advanced NSCLC patients with EGFR mutation who underwent next-generation sequencing (NGS) prior to treatment. The combination group included 50 patients who received the regimen of EGFR-TKI combined with chemotherapy, while the EGFR-TKI monotherapy group included 67 patients treated with TKI only. The primary endpoint of this study was progression-free survival (PFS); the secondary endpoints were overall survival (OS), response rate, and toxicity.

**Results:**

The median PFS was significantly longer in the combination group than in the EGFR-TKI monotherapy group (19.00 months [95% CI, 14.67–23.33] vs. 11.70 months [95% CI, 10.81–12.59], *p* < 0.001). Subgroup analysis showed a similar trend of results. The median OS was not reached in the combination group and was 38.50 (95% CI, 35.30–41.70) months in the EGFR-TKI monotherapy group (*p* = 0.586). Patients in the combination group were more likely to experience adverse events, most of which showed the severity of grade 1 or 2. T790M mutation remains the main reason for acquired resistance, and the frequency of T790M mutation was similar between the two groups (*p* = 0.898).

**Conclusions:**

Compared with EGFR-TKI monotherapy, EGFR-TKI combined with chemotherapy significantly improved PFS in advanced NSCLC patients with EGFR mutation, with acceptable toxicity.

**Supplementary Information:**

The online version contains supplementary material available at 10.1186/s12885-021-08291-9.

## Background

GLOBOCAN 2018 shows that lung cancer remains the commonest cancer and a leading cause of cancer death worldwide [1]. Non-small cell lung cancer (NSCLC) occurs in approximately 85% of all cases [2]. In the past two decades, new technologies, like molecular and histological testing and next-generation sequencing (NGS), have greatly reformed the treatment of NSCLC. A consensus has been made that the epidermal growth factor receptor (EGFR) is implicated in the pathogenesis of NSCLC. An increasing number of studies reported that for EGFR-mutant patients, EGFR- tyrosine kinase inhibitors (TKIs) brings a higher objective response rate (ORR) and longer progression-free survival (PFS) compared to traditional chemotherapy [3–8]. These studies have led to the era of personalized therapy. For NSCLC patients harboring EGFR mutation, EGFR-TKIs have been standardized into the first-line treatment.

Although targeted therapies have achieved much for NSCLC patients, challenges remain [9]. The most noteworthy is drug resistance, including initial resistance and acquired resistance. Different mechanisms of acquired resistance to EGFR-TKIs have been reported. The acquired resistance to first-generation TKIs is primarily caused by the second point mutation, with a threonine-to-methionine acid change at position 790 (T790M) of exon 20 [10]. Other mechanisms include amplification in HER2, MET, EGFR, or mutations in MET, BRAF, PIK3CA, and SCLC transformation epithelial-to-mesenchymal transition [9, 11]. The results of AURA3 have proved the efficacy of osimertinib, a third-generation EGFR-TKI that is selective for original sensitizing and T790M mutations in NSCLC patients [12]. Additionally, the results of FLAURA indicated that the efficacy of osimertinib was superior to standard TKIs as the first-line treatment of EGFR mutation-positive advanced NSCLC [13, 14]. Based on this, osimertinib has been the preferred recommended as the first-line treatment regimen for advanced NSCLC patients with EGFR mutation regimen by the NCCN guidelines. However, first-generation TKI is still recommended by NCCN and CSCO clinical practice guidelines in oncology. And because of its long history of use and lower price, it is still widely used in clinical practice. Extending the survival time of patients and overcoming or delaying acquired drug resistance has become a new problem. EGFR-TKI combined with chemotherapy, immunotherapy, anti-angiogenesis, radiotherapy, and other treatments may solve this problem.

As we all know, pemetrexed is a multitargeted antifolate that inhibits multiple enzymes involved in folate metabolism, including thymidylate synthase (TS) [15]. In addition, studies in vitro and vivo suggested that first-generation TKI could also down-regulate TS at mRNA and protein levels [16–18]. The synergistic effect of TKI and pemetrexed provides a molecular foundation for the use of TKI plus chemotherapy. Herein, we retrospectively assessed the efficacy of EGFR-TKI alone or in combination with chemotherapy as first-line therapy for treatment-naïve advanced NSCLC patients.

## Methods

### Patients

We conducted retrospective research of NSCLC patients who were treated at the First Affiliated Hospital of Nanjing Medical University between November 2014 and August 2019. All of the NSCLC patients were histopathologically confirmed, and advanced NSCLC was defined as stage IIIb/c and IV according to the AJCC (American Joint Committee on Cancer) Cancer Staging Manual (8th edition). Inclusion criteria: (1) pathologically diagnosed NSCLC; (2) underwent NGS prior to treatment, and genome sequencing confirmed EGFR mutation (primarily exon 21 L858R point mutation or exon 19 deletion); (3) first-line treatment was first-generation TKI or TKI in combination with chemotherapy; (4) age ≥ 18; (5) Eastern Cooperative Oncology Group (ECOG) performance status was ≤2; (6) a life expectancy of longer than 3 months; and (7) without other malignant tumor histories. The study was conducted according to the Declaration of Helsinki and approved by the First Affiliated Hospital of Nanjing Medical University Ethics Committee. All participants included in the study signed written informed consent before treatment.

### Treatment

Patients assigned to the monotherapy group received EGFR-TKI therapy (gefitinib 250 mg po qd, icotinib 125 mg po tid or elortinib 150 mg po qd). Patients assigned to the combination group received EGFR-TKI therapy combined with chemotherapy (cisplatin or carboplatin plus pemetrexed, or pemetrexed alone). After 6 cycles of chemotherapy, EGFR-TKI was combined with pemetrexed as maintenance therapy. Treatment continued until progressive disease (PD), unacceptable toxicity, or withdrawal of consent.

### Assessment of efficacy and adverse events

The primary endpoint of this study was progression-free survival (PFS); secondary endpoints included overall survival (OS), response rate, and toxicity. PFS was defined as the period from the start of treatment to disease progression, death or the last follow-up, and OS was defined as the time from the start of treatment to death or the deadline of follow-up. Best response time was defined as the time from the start of treatment to tumor no longer shrinks, disease progression, death, or the last follow-up. The tumor response rate was expressed with objective response rate (ORR) and disease control rate (DCR). RECIST 1.1 was used to evaluate the tumor response. Tumor status was assessed every two cycles during chemotherapy for patients in the combination group, and for patients received EGFR-TKI monotherapy was assessed every 2 months or at overt signs of progression. Adverse events (AEs) were assessed according to Common Terminology Criteria for Adverse Events of the National Cancer Institute (CTCAE 4.0) and were rated from grades 1 to 5.

### Statistical analysis

The chi-squared test was used for comparisons of ORR and DCR intergroup at a significance level of 5% (a = 0.05, two-sided). PFS and OS were analyzed by the Kaplan-Meier method. The log-rank test was utilized to compare the significance between groups, while the Cox proportional hazards model was used for the multivariate survival analysis. *p*-values of < 0.05 (*p* < 0.05) were considered statistically significant. SPSS software (version 20.0; SPSS Inc.), RStudio (Version 1.2.1335; RStudio, Inc.) and Adobe Illustrator 2020 were used for all statistical analysis and create the graphics.

## Results

### Patients and clinical characteristics

A total of 117 patients were retrospectively analyzed in this retrospective study. Patients received first-generation EGFR-TKI or plus chemotherapy. Among them, 50 patients in combination group (T + C) received chemotherapy plus first-generation EGFR-TKI, while the other 67 patients in the monotherapy group (T) received EGFR-TKI alone. From January 2017 to August 2019, 89 patients received EGFR-TKI monotherapy in our center, 22 of them were excluded for various reasons, and 67 of them were eventually included in this study. Of the 22 excluded cases, 13 were due to unknown EGFR mutation status, 5 were due to less than 3 months of treatment or less than 3 months of follow-up, 2 due to ECOG score greater than 2, 1 due to squamous cell carcinoma, and 1 due to combined thyroid cancer. In the combination group, three patients received pemetrexed monotherapy, all of whom were elderly. They chose pemetrexed monotherapy because of the heavy tumor burden and advanced age. The patients’ clinical characteristics were summarized in Table 1, including age, sex, histology, ECOG PS, smoking history, EGFR mutation, brain metastasis, stage, cardiovascular and metabolic. There was no significant difference in clinical characteristics between the two groups.

**Table 1:** Characteristics of all patients

Characteristic	T + C (*n* = 50)	T (*n* = 67)	*p*
**Age (years)**			0.294
Median (range)	59(36–81)	61(40–84)	
**Sex**			0.506
Male	24(48.00%)	28(38.27%)	
Female	26(52.00%)	39(61.73%)	
**Histology**			1.000
Adenocarcinoma	50(100.00%)	67(100.00%)	
**ECOG PS**			0.835
0–1	49(98.00%)	66(98.77%)	
2	1(2.00%)	1(1.23%)	
**Smoking history**			0.986
Nerve	35(70.00%)	47(72.84%)	
Former	15(30.00%)	20(27.16%)	
**EGFR mutation**			0.321
Exon 19 deletion	26(52.00%)	41(55.56%)	
L858R	21(42.00%)	23(38.27%)	
Others	3(8.00%)	3(6.17%)	
**Brain metastasis**			0.345
Yes	9(18.00%)	17(23.46%)	
No	41(82.00%)	50(76.54%)	
**Stage**			0.713
IIIb/IIIc	3(6.00%)	3(3.70%)	
IV	47(94.00%)	64(96.30%)	
**Cardiovascular**			
Hypertension	15(30.00%)	21(31.34%)	0.877
Coronary heart disease	0(0.00%)	1(1.23%)	0.388
Cerebrovascular disease	0(0.00%)	2(2.99%)	0.220
**Metabolic**			
Diabetes	6(12.00%)	6(8.96%)	0.593
Hyperlipidemia	2(4.00%)	1(1.23%)	0.398
Hyperuricemia	0(0.00%)	1(1.23%)	0.388

### Tumor response

After two cycles / 2 months of treatment, the response rate was evaluated. Of the 50 patients in the combination group, 1 achieved complete response (CR), 38 achieved partial response (PR), 10 achieved stable disease (SD), 1 achieved PD, resulting in an ORR of 78.00% and DCR of 98.00%. Of the 67 patients in the EGFR-TKI monotherapy group, 43 achieved PR, 22 achieved SD, 2 achieved PD, and no one achieved CR, resulting in an ORR of 64.18% and DCR of 97.01% (Table S1). As shown in Table S1, the ORR was slightly higher in the combination group than in the EGFR-TKI monotherapy group, but there was no statistically significant difference (*p* = 0.108). We also evaluate the best response time. The median best response time was 132 days (95% CI, 113.54–150.46) in the combination group and 116 days (95% CI, 85.06–146.94) in the EGFR-TKI monotherapy group, but there was no statistically significant difference (*p* = 0.651) (Fig S1).

### Survival analysis

**Fig. 1:**
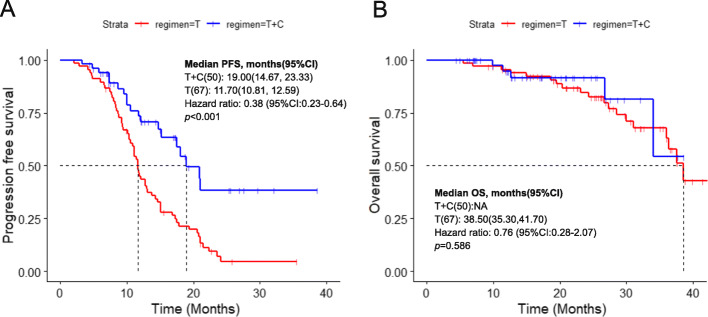
Progression-free survival (**a**) and overall survival (**a**) in two groups. T + C, EGFR-TKI combined with chemotherapy; T, EGFR-TKI monotherapy; PFS, progression-free survival; OS, overall survival

As of March 2020, 79 patients (67.52%) had reached the endpoint of disease progression or death, and the median follow-up time was 26.27 months. The median PFS (mPFS) was 19.00 months (95% CI, 14.67–23.33) in the combination group and 11.70 months (95% CI, 10.81–12.59) in the EGFR-TKI monotherapy group, and the difference was statistically significant (*p* < 0.001) (Fig. 1a). The median OS (mOS) was not reached in the combination group and no difference in OS was identified at the time of this analysis (NA vs. 38.50 months, *p* = 0.586, Fig. 1b). We also used Kaplan-Meier curve to present 1- and 2-year survival, but there was no statistical difference (Fig S2a,b). In the combination group, 39 patients were followed up for more than 1 year or died within 1 year, of which two patients died within 1 year, so the 1-year survival rate was 94.87% (37/39). The number of effective follow-ups for 1–2 years was 12, and one died within 1–2 years, so the 2-year survival rate was 88.10% (11/12 × 37/39). Similarly, in the TKI monotherapy group, the 1-year survival rate was 96.82% (61/63) and 82.09% (39/46 × 61/63). There was no significant difference between the two groups (*p* = 0.303) (Fig S2c). As the overall survival data was not sufficient enough, further analysis was not performed.

### EGFR mutation site analysis

**Fig. 2:**
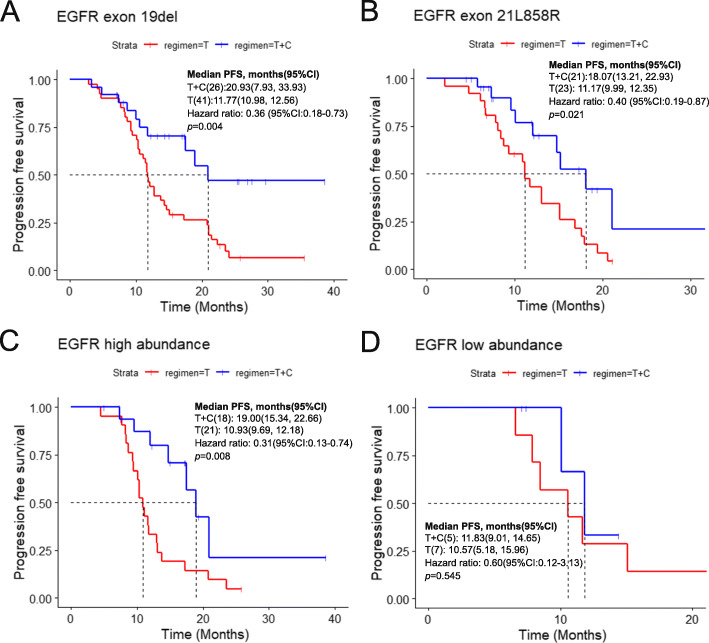
Progression-free survival in different subgroups **a** EGFR exon 19 deletion subgroup; **b** EGFR exon 21L858R subgroup; **c** EGFR high abundance subgroup; **d** EGFR low abundance subgroup. T + C, EGFR-TKI combined with chemotherapy; T, EGFR-TKI monotherapy; PFS, progression-free survival; OS, overall survival

In all patients, approximately 57.26% (*n* = 67) had an exon 19 deletion (19 del), while 37.61% (*n* = 44) had an exon 21 L858R mutation (21 L858R) in the EGFR gene. For the patients with EGFR exon 19 deletion, the mPFS was 20.93 months (95% CI, 7.93–33.93) in the combination group and 11.77 months (95% CI, 10.98–12.58) in the EGFR-TKI monotherapy group (*p* = 0.004) (Fig. 2a). The mPFS for patients harboring L858R point mutation was 18.07 months (95% CI, 13.21–22.93) in the combination group and 11.17 months (95% CI, 9.99–12.35) in the EGFR-TKI monotherapy group (*p* = 0.021) (Fig. 2b).

### EGFR mutation abundance analysis

In this study, abundance data was available in 51 patients, including 23 were in the combination group and 28 were in the EGFR-TKI monotherapy group. We explored the relationship between the EGFR mutation abundance and the efficacy of EGFR TKI combination with or without chemotherapy. The cutoff value of mutation abundance was set as 4.9% for exon 19 deletion and 9.5% for exon 21 L858R [19]. The cutoff value of ctDNA abundance from plasma was set as 2% for exon 19 deletion and 5% for exon 21 L858R [19]. Of all 51 patients, 39 patients harbored high abundance EGFR mutation and 12 had low abundance mutation. Among patients with high EGFR mutation abundance, the mPFS was 19.00 (95% CI, 15.34–22.66) months in the combination group and 10.93 (95% CI, 9.69–12.18) months in the EGFR-TKI monotherapy group (*p* = 0.008) (Fig. 2c). However, among patients with low EGFR mutation abundance, mPFS was 11.83 months (95% CI, 9.01–14.65) vs. 10.57 months (95% CI, 5.18–15.96), and the difference was not significant (*p* = 0.545) (Fig. 2d).

### Subgroup analysis

**Fig. 3:**
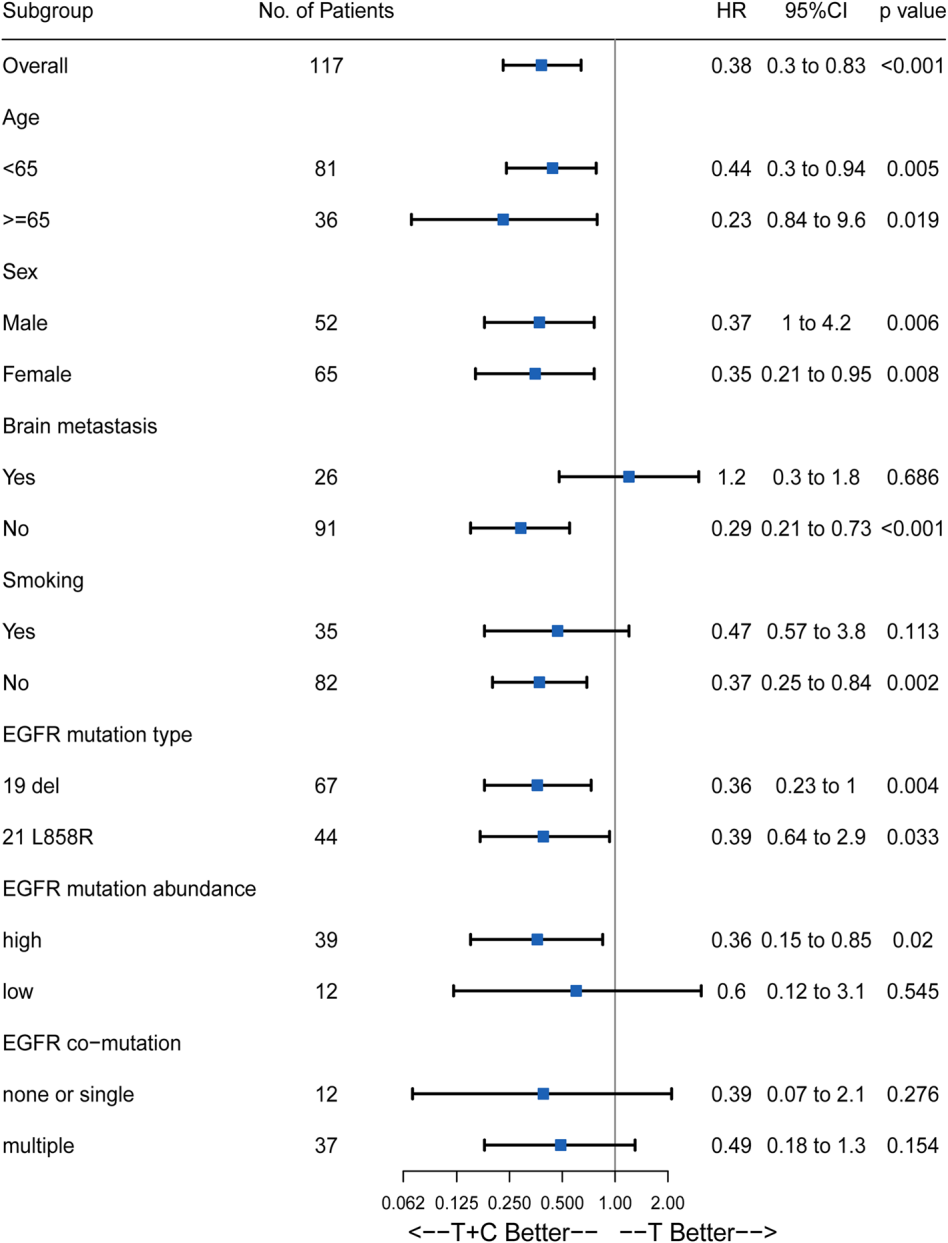
Subgroup analysis of progression-free survival HR: Hazard ratios; Del 19 del, exon 19 deletion; 21 L858R, exon 21 point mutation;T + C, EGFR-TKI combined with chemotherapy; T, EGFR-TKI monotherapy

Subgroup analysis was conducted to screen out the intended population. The Cox regression model was used to calculate hazard ratios. Figure 3 showed that the results of the subgroup analysis were basically consistent with the above-mentioned result. Most patients may obtain clinical benefits from the regimen of TKI combined with chemotherapy. But for patients with brain metastasis, T + C did not show a significant advantage and the risk of progression was 1.2 times higher in the combination group than that in the TKI monotherapy group (*p* = 0.686; HR = 1.2; 95% CI, 0.3–1.8). Significant difference was also not deserved in subgroups of none or single EGFR co-mutation and multiple (≥2) co-mutation (*p* = 0.276, HR = 0.39, 95% CI (0.07–2.1) in none or single co-mutation subgroup and *p* = 0.154, HR = 0.49, 95% CI, (0.18–1.3) in multiple co-mutation subgroup).

### After progression

**Fig. 4:**
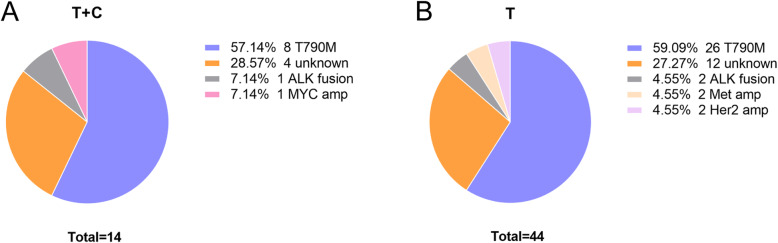
The resistance mechanism clarified by the patient and the frequency of T790M in two groups **a** EGFR-TKI combined with chemotherapy; **b** EGFR-TKI monotherapy

Disease progression was inevitable. Most patients experienced asymptomatic progression, and the site of progression was shown in Fig S3. Among these patients, only three patients in the combination group had bone pain, dizziness, and headache, while seven patients in the TKI monotherapy group had bone pain, hemoptysis, and dizziness. During the treatment, seven patients in the combined group received palliative radiotherapy, 3 received brain radiotherapy, and 4 received bone radiotherapy. In the TKI monotherapy group, 12 patients received palliative radiotherapy, 5 received brain radiotherapy, and 7 received bone radiotherapy. Considering the impact of drug resistance on treatment, re-biopsy, and NGS-based testing were made. T790M was detected in 59.09% (26/44) of patients in the TKI monotherapy group and 57.14% (8/14) in the combination group, and the results suggested that there was no statistical difference in the frequency of T790M mutation (*p* = 0.898). Acquired resistance also involved Her2 amplification, Met amplification, ALK fusion, and Myc amplification (Fig. 4a, b). Of the 117 patients, 34 patients obtained T790M mutation after progression, and 27 patients received third-generation EGFR-TKIs, of which 7 were in the combination group, and 20 were in the EGFR-TKI monotherapy group.

### AEs

The details about the AEs were shown in Table 2. Skin rash was the most common (64.00% in the combination group and 70.15% in the EGFR-TKI monotherapy group) (*p* = 0.804), followed by elevated liver enzymes (62.00% in the combination group and 50.74% in the EGFR-TKI monotherapy group, *p* = 0.228). The other AEs observed in the TKI monotherapy group included diarrhea (35.82%), mucositis (13,43%), constipation (10.45%), and nausea/vomiting (10.45%). Meanwhile, hematologic toxicities were more common in the combination group, such as leukopenia/neutropenia (44.00% vs. 7.46%, *p* = 0.000), anemia (38.00% vs. 8.96%, *p* = 0.000), thrombocytopenia (34.00% vs. 8.96%, *p* = 0.001). The patients in the combination group were more likely to develop AEs, most of which showed the severity of grade 1 or 2. In 6 patients (8%) in the combination group, chemotherapy or TKI was delayed as a result of toxicities. In the EGFR-TKI monotherapy group, 5 (7.46%) patients experienced short-lived delays due to AEs. No drug-related interstitial lung disease or deaths were observed.

**Table 2:** Adverse events

Toxicity	T + C (*n* = 50)		T (*n* = 67)	
	Any	≥3	Any	≥3
Skin rash	32(64.00%)	2(4.00%)	47(70.15%)	2(2.99%)
Elevated AST / ALT	31(62.00%)	6(12.00%)	34(50.74%)	7(10.45%)
Diarrhoea	19(38.00%)	1(2.00%)	24(35.82%)	1(1.49%)
Leukopenia / Neutropenia	22(44.00%)	7(14.00%)	5(7.46%)	0(0.00%)
Anaemia	19(38.00%)	5(10.00%)	6(8.96%)	0(0.00%)
Thrombocytopenia	17(34.00%)	6(12.00%)	6(8.96%)	0(0.00%)
Nausea / Vomiting	14(28.00%)	2(4.00%)	7(10.45%)	0(0.00%)
Fatigue	16(32.00%)	2(4.00%)	5(7.46%)	0(0.00%)
Anorexia	15(30.00%)	2(4.00%)	710.45%)	1(1.49%)
Mucositis	12(24.00%)	1(1.23%)	9(13.43%)	1(1.49%)
Constipation	10(20.00%)	0(0.00%)	7(10.45%)	0(0.00%)

## Discussion

Many clinical trials have confirmed EGFR-TKIs as the standard first-line therapy for advanced NSCLC patients with EGFR sensitive mutations. Apart from their significant benefits, TKIs inevitably trigger acquired drug resistance [20]. Blakely CM et.al pointed out that tumor genomic complexity increased with the prolongation of EGFR-TKIs treatment, a change that, sometimes along with the co-mutation of other genes, can promote tumor development or limits EGFR inhibitor response [21]. In addition, the presence of intratumor heterogeneity and resistant subclones may also discount the efficacy of TKI [22]. Meanwhile, a retrospective cohort study verified that genetic co-alterations negatively affect the response and survival of patients with EGFR mutation [23]. All these findings have laid a theoretical foundation for the use of combination therapy. Clinical studies had been carried out to explore the feasibility of TKI combined with other treatments, such as chemotherapy and vascular endothelial growth factor (VEGF) inhibitors [24, 25]. Some of them have yielded encouraging results. Here, we show the results of a real-world study of EGFR-TKI in combination with chemotherapy.

Previous clinical studies have found that TKI combined with chemotherapy is superior to EGFR-TKI monotherapy in PFS. JMIT, the first randomized study to examine pemetrexed plus EGFR-TKI therapy as first-line treatment for advanced NSCLC patients with activating EGFR mutations, showed that the combination therapy improved PFS compared with TKI monotherapy [26]. Similarly, phase III randomized trials in Japanese and Indian population (NEJ009) also proved that a combination (pemetrexed+carboplatin+gefitinib), compared to single gefitinib, significantly prolonged PFS and OS in NSCLC patients with EGFR mutations [27, 28]. However, many clinical studies failed to find significant improvement, which may be explained by the inappropriate inclusion criteria [29]. In this study, the median PFS of patients reached 19.00 months in the combination group, but only 11.70 months in the TKI monotherapy group, thus confirming the superiority of the combination therapy mentioned before. Furthermore, the median PFS in both groups are close to those previously reported [29–31].

Biology varies with the EGFR mutation subtype in patients treated with EGFR-TKI therapy [32]. Therefore, we explored the efficacy of treatment regimens administered according to EGFR mutation subtypes. The results showed that the mPFS was longer in the combination group than in the TKI monotherapy group, regardless of whether the patient harbored EGFR exon 19 deletion or exon 21 L858R point mutation. And the results were consistent with previous studies [26].

We also explored the relationship between the abundance of EGFR mutations and the efficacy of EGFR-TKI with or without chemotherapy. Previous studies had reported that the abundance of EGFR mutation was significantly associated with the objective response to EGFR TKIs, and the mPFS in the high abundance group was significantly longer than that in the low abundance group [19, 33, 34]. The difference in EGFR mutation abundance may be caused by intratumoral heterogeneity [19]. For example, in patients with a low abundance of EGFR mutations, tumor clones without EGFR mutations may dominate in the primary tumors [19]. The result of this study also suggests that patients with a high EGFR mutation abundance may benefit more from the combination of TKI with chemotherapy. The superiority of combination therapy may be due to the synergy between TKI and pemetrexed, and studies in vitro and vivo have proved [16–18]. Besides, we believe that combination therapy can also prolong the PFS of patients with low-abundance EGFR mutations, but the difference is not significant due to the small sample size in this study. Moreover, previous studies have confirmed that EGFR-TKI plus chemotherapy could significantly improve PFS and OS in patients with low-abundance mutations as first-line treatment [35]. But in our study, this improvement was not significant, which may be due to the small sample size.

This study also showed no significant difference in the ORR and DCR between the two groups, which is basically consistent with previous findings [26, 35]. However, many studies have still shown the treatment regimen of TKI combined with chemotherapy was associated with a higher response rate [27, 28]. There was even a study reporting that a greater depth of response was associated with longer PFS and OS [36]. Additionally, we evaluated the best response time and found that the best response time of TKI monotherapy was 116 days, which was close to those previously reported [37]. When compared the best response time between the two groups, the statistical difference did not show, which was consistent with the results of tumor response rate. Deeper research is also needed to validate these findings. The results of the TKI monotherapy group initially indicated that OS reached 38.50 months, which was similar with privous studies [28]. Unfortunately, we had not yet been able to obtain OS data for the combined treatment group.

The results of the subgroup analysis indicated that the combined therapy regimen was superior to EGFR TKI monotherapy for most patients. And the combined therapy regimen exerted a better efficacy on the young, females, never-smokers, and those without brain metastasis and high EGFR mutation abundance. This finding was consistent with the precedents advocating the superiority of TKI plus therapy over gefitinib in any subgroup [26–28]. Interestingly, the intended population happens to be those who respond well to TKI, which may also be explained by the EGFR mutation rate and mutation abundance.

T790M mutation, a second EGFR mutation, provokes acquired resistance in about half cases taking first-generation TKIs [38]. FLAURA trial demonstrated that the third-generation TKI osimertinib had better efficacy in patients with the T790M mutation. The proportions of patients with T790M at post-progression patients in this study were consistent with those in previous studies, and no significant difference was observed between treatment groups. Our results revealed that chemotherapy plus TKI does not reduce the frequency of EGFR T790M mutations, which means that the third-generation EGFR TKI osimertinib can still be used after progression. The result hinted that conservative chemotherapy plus TKI might delay the emergence of TKI resistance, and previous studies had also proved that the combination of gefitinib and pemetrexed prevented TKI resistance mediated by T790M mutation or epithelial-to-mesenchymal transition (EMT) in EGFR-mutant NSCLC cell lines and xenograft models [39]. On the other hand, the superiority of combination therapy may result from the synergistic effect of TKI and pemetrexed in down-regulating TS and arresting the cell cycle [16, 18]. TKI combined with anti-angiogenesis therapy was also an alternative to overcome drug resistance. RELAY, a randomized phase 3 trial, reported that ramucirumab plus erlotinib demonstrated superiority in prolonging PFS over placebo plus erlotinib (19.4 months vs. 12.4 months, *p* < 0.0001) [25]. The PFS achieved by TKI combined with chemotherapy in our study was similar to that by TKI combined with ramucirumab in RELAY, and the OS could not be compared due to the immature data. A recent study suggested that the frequency of EGFR T790M mutations seems reduced in patients treated with EGFR-TKI plus bevacizumab than EGFR-TKI monotherapy [24]. The effect of anti-angiogenesis therapy on the frequency of T790M is not conclusive, so more data are needed to define the population suitable to each regimen.

In this study, we explored the relationship between mutation subtype and EGFR mutation abundance and therapeutic response, which is a significant innovation compare with other previous clinical trials [26–28]. Additionally, we studied comparative resistance mechanisms in TKI monotherapy and combination and investigated the medications of patients after progression. Last but not least, this study was retrospective research in a real-world setting, the results obtained were more in line with the actual clinical situation and are real world. The results have important clinical significance. This real-life analysis also has several limitations. A limitation in this study is that less than half of the patients had mutation abundance data, making it difficult to analyze the relationship between EGFR abundance and the efficacy of treatment regimens. Another limitation is the insufficiency of OS data. In addition, this retrospective study was conducted using data from real-world settings, so it cannot be monitored rigorously like a randomized controlled trial. Finally, the small sample size, retrospective nature, and heterogeneity of treatment regimens were also limitations in our study.

## Conclusions

In conclusion, TKI combined with chemotherapy is superior over EGFR-TKI monotherapy in prolonging mPFS, for the most subgroup of advanced NSCLC patients harboring the EGFR mutation. PFS of patients with high EGFR mutation abundance in the combination group was significantly longer than that in the EGFK-TKI monotherapy group, but there was no significant difference in PFS among patients with low mutation abundance. EGFR-TKI combination with chemotherapy may delay acquired resistance against first-generation EGFR-TKI, which requires further research.

## Supplementary Information

**Additional file 1: Fig. S1.** Best response time in different subgroups. T + C, EGFR-TKI combined with chemotherapy; T, EGFR-TKI monotherapy. **Fig. S2.** 1-year survival (a) (start the Y-axis with 90%) and 2-year survival (b) (start the Y-axis with 70%) in two groups; (c) 1-year and 2-year OS rates in two groups. T + C, EGFR-TKI combined with chemotherapy; T, EGFR-TKI monotherapy; OS, overall survival. **Fig. S3.** The site of progression in two groups. T + C, EGFR-TKI combined with chemotherapy; T, EGFR-TKI monotherapy. **Table S1.** Tumor response.

## Data Availability

The datasets used or analyzed during the current study are available from the corresponding author on reasonable request.

## Abbreviations

EGFR: Epidermal growth factor receptor
TKI: Tyrosine kinase inhibitors
NSCLC: Non-small cell lung cancer
NGS: Next-generation sequencing
PFS: Progression-free survival
OS: Overall survival
ORR: Objective response rate
TS: Thymidylate synthase
AJCC: American Joint Committee on Cancer
ECOG: Eastern Cooperative Oncology Group
PD: Progressive disease
DCR: Disease control rate
AE: Adverse events
CTCAE: Criteria for Adverse Events of the National Cancer Institute
CR: Complete response
PR: Partial response
SD: Stable disease
VEGF: Vascular endothelial growth factor
