# Supplementary material for: Efficacy and acquired resistance of EGFR-TKI combined with chemotherapy as first-line treatment for Chinese patients with advanced non-small cell lung cancer in a real-world setting
Source: BMC Cancer. 2021 May 25;21:602. doi: 10.1186/s12885-021-08291-9 (PMC8152122; doi:10.1186/s12885-021-08291-9)
Supplement: Supplementary file 1 — Additional file 1: Fig. S1. Best response time in different subgroups. T + C, EGFR-TKI combined with chemotherapy; T, EGFR-TKI monotherapy. Fig. S2. 1-year survival (a) (start the Y-axis with 90%) and 2-year survival (b) (start the Y-axis with 70%) in two groups; (c) 1-year and 2-year OS rates in two groups. T + C, EGFR-TKI combined with chemotherapy; T, EGFR-TKI monotherapy; OS, overall survival. Fig. S3. The site of progression in two groups. T + C, EGFR-TKI combined with chemotherapy; T, EGFR-TKI monotherapy. Table S1. Tumor response. [file 12885_2021_8291_MOESM1_ESM.docx]

**Supplementary materials**

**Fig. S1** Best response time in different subgroups. T+C, EGFR-TKI combined with chemotherapy; T, EGFR-TKI monotherapy


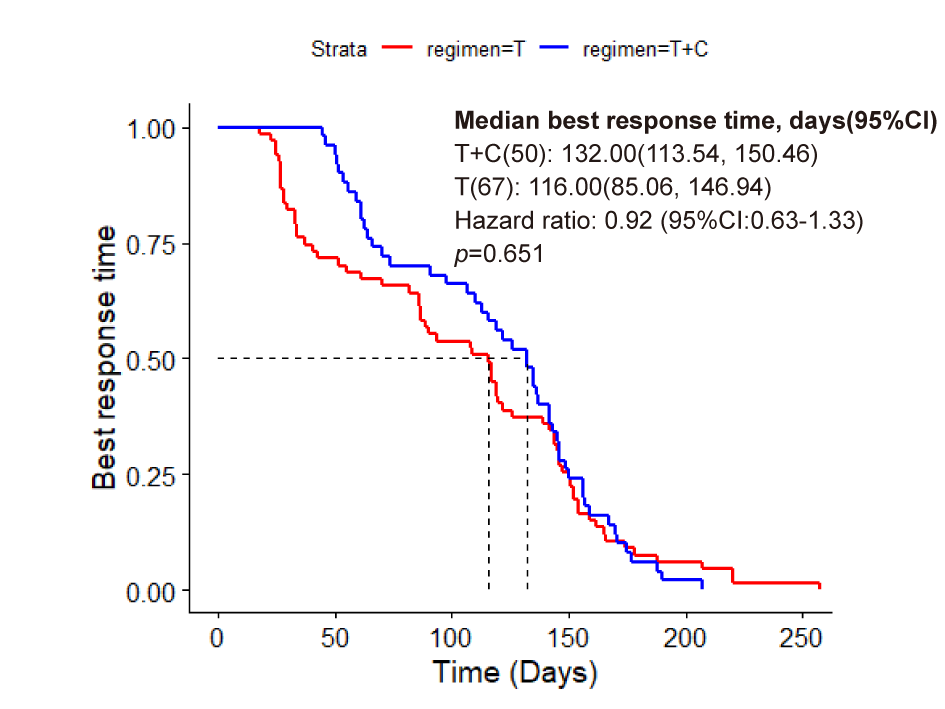


**Fig. S2** 1-year survival (a) (start the Y-axis with 90%) and 2-year survival (b) (start the Y-axis with 70%) in two groups; (c) 1-year and 2-year OS rates in two groups. T+C, EGFR-TKI combined with chemotherapy; T, EGFR-TKI monotherapy; OS, overall survival


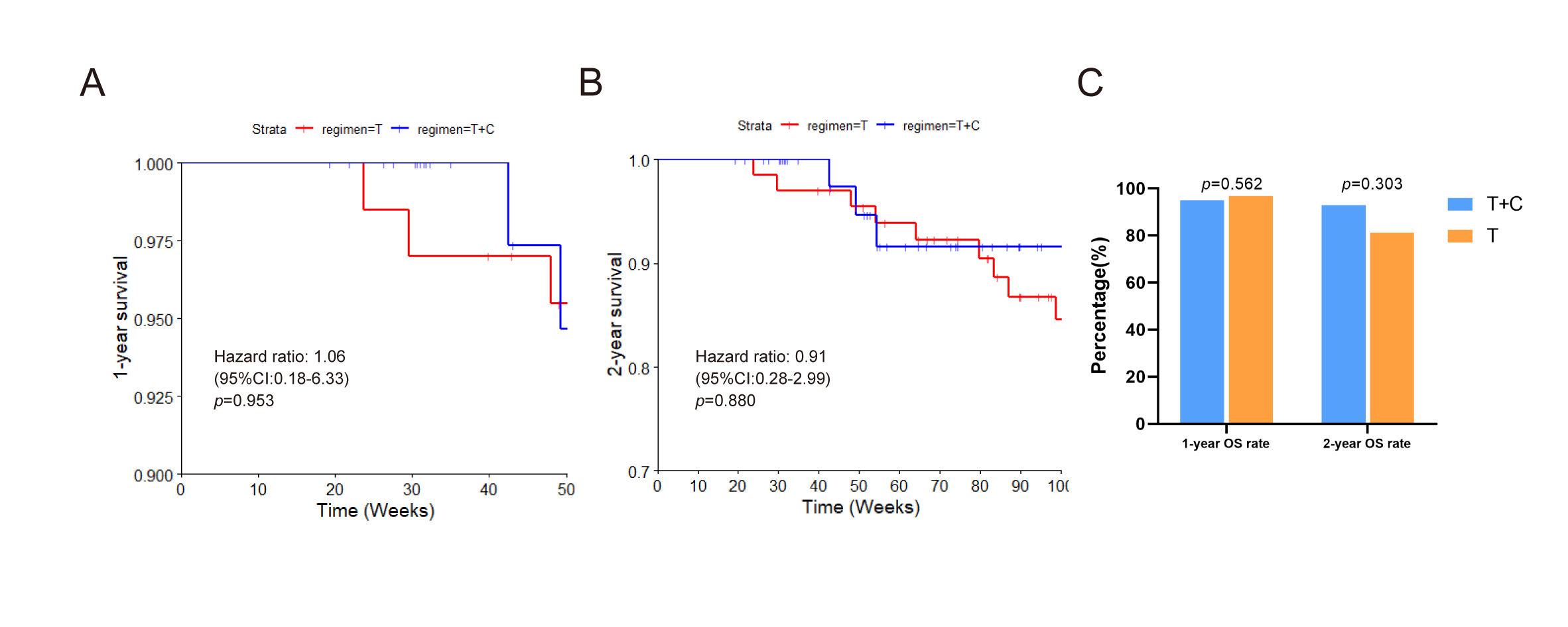


**Fig S3** The site of progression in two groups. T+C, EGFR-TKI combined with chemotherapy; T, EGFR-TKI monotherapy


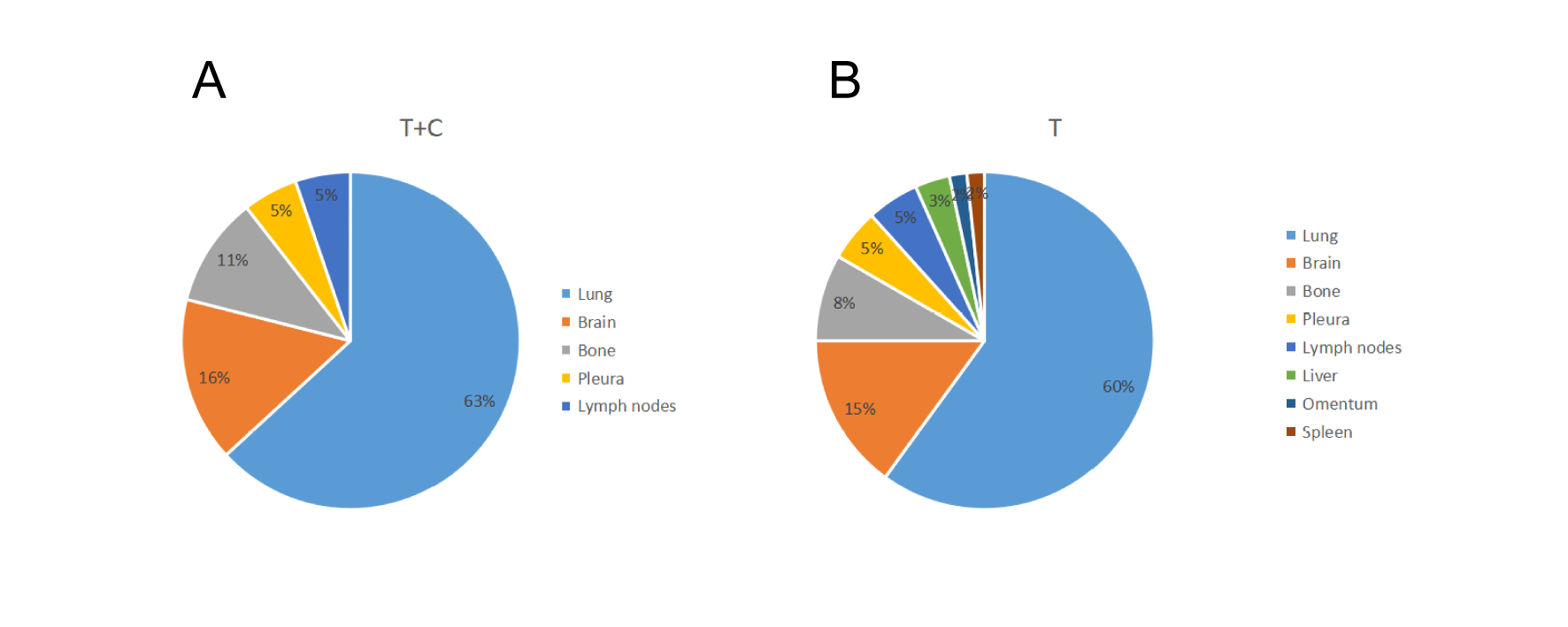


**Table S1 Tumor response**

| Response | T+C (n=50) | T(n=67) | *p* |
| --- | --- | --- | --- |
| CR | 1(2.00%) | 0(0.00%) | 0.247 |
| PR | 38(76.00%) | 43(64.18%) | 0.172 |
| SD | 10(20.00%) | 22(32.84%) | 0.125 |
| PD | 1(2.00%) | 2(2.98%) | 0.740 |
| ORR (CR+PR) | 39(78.00%) | 43(64.18%) | 0.108 |
| DCR (CR+PR+SD) | 49(98.00%) | 65(97.01%) | 0.740 |
